# Open-source convolutional neural network to classify distal radial fractures according to the AO/OTA classification on plain radiographs

**DOI:** 10.1007/s00068-025-02931-6

**Published:** 2025-07-21

**Authors:** Koen D. Oude Nijhuis, Jasper Prijs, Britt Barvelink, Hans van Luit, Yang Zhao, Zhibin Liao, Ruurd L. Jaarsma, Frank F. A. IJpma, Mathieu M. E. Wijffels, Job N. Doornberg, Joost W. Colaris, Machine Learning Consortium, Charlotte Laane, Olga Canta, Sanne Hoeksema, Kaan Aksakal, Haras Mhmud, Paul Jutte, Wouter Mallee, Andrew Duckworth, Niels Schep, Ran Hendrickx, Anne-Eva Bulstra, Lente Dankelman

**Affiliations:** 1 Department of Orthopedic Surgery, University Medical Centre Groningen and Groningen University, Groningen, The Netherlands; 2 Department of Trauma Surgery, University Medical Centre Groningen and Groningen University, Hanzeplein 1, 9713PZ Groningen, The Netherlands; 3 Department of Orthopedic Surgery, Flinders University and Medical Centre, Adelaide, South Australia Australia; 4 Department of Orthopedics and Sports Medicine, Erasmus University Medical Centre, Rotterdam, The Netherlands; 5 Australian Institute for Machine Learning, Adelaide, Australia; 6 Trauma Research Unit Department of Surgery, Erasmus MC, University Medical Center Rotterdam, P.O. Box 2040, Rotterdam, 3000 CA The Netherlands

**Keywords:** Wrist fractures, Artificial intelligence, Diagnostic accuracy, Trauma

## Abstract

**Purpose:**

Convolutional Neural Networks (CNNs) have shown promise in fracture detection, but their ability to improve surgeons' inconsistent fracture classification remains unstudied. Therefore, our aim was create and (externally) validate the performance of an open-source CNN algorithm to classify DRFs according to the AO/OTA classification system?

**Methods:**

Patients with postero-anterior, lateral and oblique radiographs were included. Radiographs were classified according to the AO/OTA-classification and were used to train a CNN algorithm. The algorithm was tested on an internal and external validation set (two other level 1 trauma centers), with the DRFs classified by three independent surgeons.

**Results:**

659 radiographs were used to train the algorithm. Internal- and external validation sets contained 190 and 188 patients, respectively. Upon internal validation, the CNN had an accuracy of 62% and an area under receiving operating characteristic curve (AUC) of 0.63–0.93 (type 2R3A 0.84, type 2R3B 0.63, type 2R3C 0.75, and no DRF 0.93). On the external validation, the algorithm has an accuracy of 61% and an AUC of 0.56–0.88 (type 2R3A 0.82, type 2R3B 0.56, type 2R3C 0.75, and no DRF 0.88).

**Conclusion:**

The presented algorithm has demonstrated excellent accuracy in classifying type 2R3A DRFs and excluding DRFs. However, poor to moderate accuracy is observed in classifying 2R3B and 2R3C DRFs according to the AO/OTA system, similar to limited surgeons’ inter-observer agreement. These results show that despite previous excellence in fracture detection, CNN-algorithms struggle with classifying; potentially showing the inherent problems with these classification systems.

## Introduction

Developing a fracture classification tool that does not suffer from inherent surgeon bias is of interest. Convolutional Neural Network (CNN) performed on the same level as clinicians in detecting fractures of the distal radius, hand, ankle, hip, and proximal humerus on plain radiographs [1–3], as also shown earlier by our research group in this journal [4]. Multiple studies showed high performance in classifying proximal humeral, hip, and knee fractures (Table 1) [3, 5–8]. Two studies have attempted classifying DRFs, but did not use traditional classification systems [9, 10]. Instead they used an extra-articular vs intra-articular classification which showed good results, and a second study did a more in-detail look at fragment displacement, joint involvement and multiple fragments, with less promising results (Table 2).

**Table 1:** Results of studies found in literature determining the performance of x-ray-based CNN algorithms in classifying non-distal radius fractures. The sensitivity, specificity, accuracy and AUC were used to describe the performance. * Data not available

Study	Classification system	Interobserver reliability (range per classification type if given by study)	Intraobserver reliability (range per classification type if given by study)	Radiographs (*n*)
Andersen et al. 1996	AO/OTA Frykman	0.64 0.34–0.36	0.57–0.70 0.40–0.61	55
Kreder et al. 1996	AO/OTA	0.68	0.67–0.86	30
Macdermid et al. 2001	AO/OTA Frykman Older	0.38 0.35 0.73	* * *	128
Jin et al. 2007	AO/OTA Frykman	0.28–0.71 0.24–0.51	0.45–0.57 0.40–0.63	43
Ploegmakers et al. 2007	AO/OTA Frykman Older Fernandez	* * * *	0.52 0.26 0.27 0.42	5
Plant et al. 2015	AO/OTA	0.39–0.66	0.53–0.75	*
van Buijtenen et al. 2015	AO/OTA	0.32–0.50	0.54–0.87	54
Jayakumar et al. 2016	AO/OTA	0.66–0.74	0.28–0.74	96
Waever et al. 2018	AO/OTA Frykman Older	0.45 0.41 0.10	0.58–0.87 0.46–0.63 0.10–0.21	*

**Table 2:** Results of studies found in literature determining the performance of x-ray-based CNN algorithms in classifying distal radius fractures. The sensitivity, specificity, accuracy and AUC were used to describe the performance

Study	Classification system	Sensitivity (95%CI)	Specificity (95%CI)	Accuracy (95%CI)	AUC (95%CI)
Tobler et al., 2021	Fragment displacement: Joint involvement: Multiple fragments:	*	*	59.7% 63.7% 78.2%	Set A: Set B 0.59: 0.92 0.62: 0.90 0.84: 0.91
Min et al., 2023	Extra-articular vs intra-articular fractures	83%	72%	81%	0.82

Although distal radius fractures (DRFs) are one of the most common fractures [11, 12], not one of the 20 classification systems has been proven reliable in terms of inter-observer agreement [13]. Several studies have shown that the reliability of the most common classification systems, such as Frykman, Older, Fernandez, and AO/OTA, consistently varies from poor to good when evaluated (Table 3) [13–22]. Studies on the most used AO/OTA classification system, showed an undesired wide spectrum of inter- and intra-observer reliability outcomes with kappa scores between 0.28–0.74 and 0.28–0.87 respectively, even for the main fracture types A, B and C. [14–22].

**Table 3:** Range of kappa values of studies determining the intra-and interobserver reliability in the literature comparing AO/OTA (types A, B, C) with Frykman, Fernandez and Older classification system of DRF on plain radiographs. * Data not available

Study	Classification system	Interobserver reliability (range per classification type if given by study)	Intraobserver reliability (range per classification type if given by study)	Radiographs (*n*)
Andersen et al. 1996	AO/OTA Frykman	0.64 0.34–0.36	0.57–0.70 0.40–0.61	55
Kreder et al. 1996	AO/OTA	0.68	0.67–0.86	30
Macdermid et al. 2001	AO/OTA Frykman Older	0.38 0.35 0.73	* * *	128
Jin et al. 2007	AO/OTA Frykman	0.28–0.71 0.24–0.51	0.45–0.57 0.40–0.63	43
Ploegmakers et al. 2007	AO/OTA Frykman Older Fernandez	* * * *	0.52 0.26 0.27 0.42	5
Plant et al. 2015	AO/OTA	0.39–0.66	0.53–0.75	*
van Buijtenen et al. 2015	AO/OTA	0.32–0.50	0.54–0.87	54
Jayakumar et al. 2016	AO/OTA	0.66–0.74	0.28–0.74	96
Waever et al. 2018	AO/OTA Frykman Older	0.45 0.41 0.10	0.58–0.87 0.46–0.63 0.10–0.21	*

Fracture classification should facilitate effective discussion about fracture characteristics and desired treatment options between healthcare professionals regarding radiographic findings. Moreover, it plays an essential role in research as it enables, 1) a standardized method to describe fractures in research, 2) a consistent method of recording in the electronic patient system, and 3) a comparison of studies using the same classifications. Furthermore, a reliable fracture classification system can provide insight into clinical decision-making [23]. For these reasons, improving intra- and interobserver reliability and minimizing variability is vital.

The aim of this study is to (externally) validate the performance of an ‘open source’ CNN to classify DRFs in postero-anterior (PA) and lateral radiographs according to the AO/OTA classification system.

## Patients/methods

### Study design

In this diagnostic imaging study an open-source CNN algorithm to classify DRFs according to the AO/OTA classification system was developed. For the training of the CNN algorithm, patients with a suspected DRF presenting to the Emergency Room of the Flinders Medical Centre (FMC), a level-1 trauma center, between the years 2016 and 2020, with PA and lateral radiographs (and oblique when present) were retrospectively included. Exclusion criteria included pathology other than DRF (not including concomitant ulnar styloid fractures), presence of epiphyseal growth plates, and poor image quality obstructing the distal radius (e.g., artifacts, noise, objects, under- or overexposure and casts that severely decrease image quality). Ethical approval was granted by the ethics committee (CALHN 13991). There are no conflicts of interest. The study was performed in accordance with the Clinical AI Research (CAIR) checklist, a guideline for AI research [24].

### Training dataset, labeling, and annotations

**Fig. 1:**
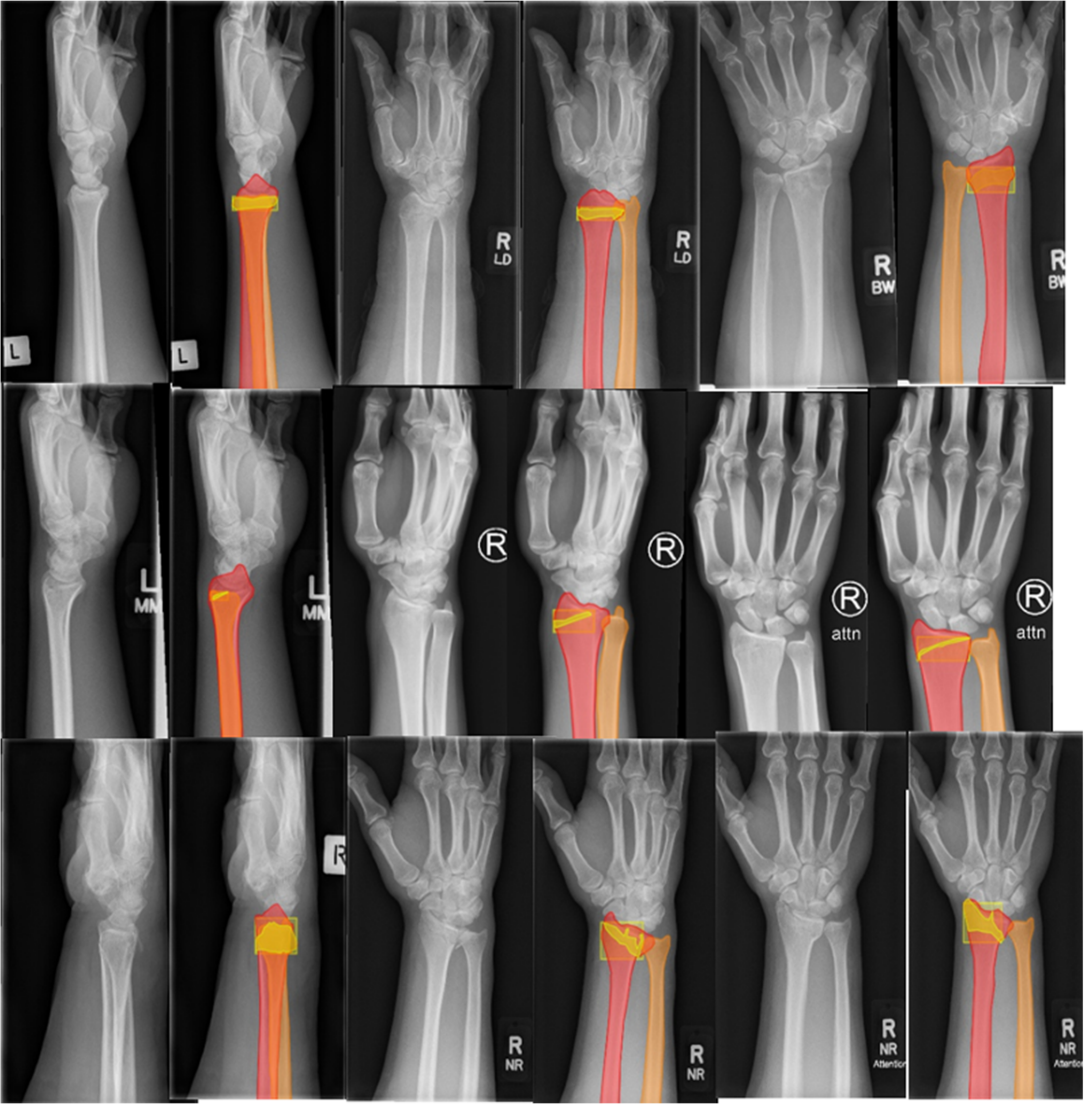
Examples of the labeling and annotation process of a type A (first row), type B (second row) and type C (third row) fracture using Labelbox software. The radius (red), ulna (orange), fractured area (yellow box) and fracture zone (yellow polygon) are indicated with different colors

The picture archiving and communication system (PACS) was searched for eligible patients with ICD-9 diagnostic codes, i.e.,"fracture"and “radius”. The radiographs were exported from PACS as Digital Imaging and Communications in Medicine (DICOM) files and subsequently anonymized with free open-source software DICOM Cleaner [PixelMed Publishing, LLC]. The DICOM files were then uploaded to an online computer vision training data platform Labelbox [25]. These images were not pre-processed. The radiographs were labeled to the presence or absence of a DRF and type of fracture according to the AO/OTA classification (2R3A for extra-articular fractures, 2R3B for partial articular fractures, 2R3C for complete articular fractures). After the image was labeled, the radius, ulna, and fracture were annotated (Fig. 1). The fracture was annotated with a rectangle and a polygon tool encompassing the fracture. Two independent reviewers performed the inclusion and exclusion and the labeling and annotations. All radiographs were checked by a senior researcher (KON, JP), under the supervision of an (orthopedic) trauma surgeon (FIJ, JD).

### Development of the algorithm

CNNs are extensively used in visual imagery analysis. These are complex multilayered networks comprised of artificial neurons [26]. The deep learning model evaluated in this study is a state-of-the-art object detection method Mask R-CNN based on Detectron2 [27]. The model consists of a backbone ResNet architecture with 50 layers and Region Proposal Network (RPN) module for bounding box proposals generation. First, we initialize the model with ImageNet pre-trained parameters. Our experiment sets the batch size to 8, and the base learning rate is initialized at 0.02. This process iterates for 6250 iterations. We repeated this experiment 5 times. All experiments are implemented with PyTorch framework on one Nvidia V100 Graphics Processing Unit (GPU). The code has been made publicly available for further training or external validation on GitHub (https://github.com/AIML-MED/DRF_Classification_Public).

### Internal validation

To evaluate performance of the algorithm, an internal validation was performed. Further patients from the FMC, the same hospital from which the training dataset was gathered, were collected in the same way as described above. Three (orthopedic) trauma surgeons (FIJ, MW, JC) reassessed all radiographs and achieved consensus on the presence or absence of a DRF and the fracture type according to the AO/OTA classification [28]. Any continued disagreements about the classification were solved during a consensus meeting.

### External validation

To test the generalizability of the algorithm, external validation was performed, meaning that the algorithm is tested with patients from external hospitals, in this case, hospitals from the other side of the world. Patients from the University Medical Center Groningen (UMCG) and the Erasmus University Medical Center (EMC), both level-1 trauma centers in the Netherlands, presented at the Emergency Room with a suspected DRF between 2015 and 2020 were collected. The same three (orthopedic) trauma surgeons (FIJ, MW, JC) independently reassessed all radiographs for external validation according to the AO/OTA classification until consensus was reached. The surgeons’ inter-observer agreement of the external validation patients was calculated.

### Statistical analysis

The performance of the algorithm is presented in several metrics. First, we assessed the algorithm's accuracy by calculating the percentage of rightly classified DRF among all cases. Then the AUC was calculated for each classification by plotting the true positive rate against the false positive rate (1-specificity). The AUC indicates how adequately the algorithm can distinguish between two groups. Lastly, the sensitivity and specificity for classifying each type of fracture are calculated. The sensitivity and specificity are the proportion of true positives and true negatives that the CNN model classifies as such. Statistical analyses were performed using SPSS version 26.0. The internal and external validation sets are seen as two different outcomes, so results are presented for both sets individually.

To present information regarding the inter-observer agreement, a Fleiss'kappa analysis was performed, which will be presented with a 95% confidence interval.

## Results

### Dataset

A total of 659 wrist radiographs from between 2016 and 2020 were included in the Flinders Medical Center record system to train the algorithm. Because of the anonymization process, it was not possible to track down the patient characteristics. A total of 188 radiographs were labeled as containing a 2R3A classified DRF, 65 radiographs as 2R3B, 62 as 2R3C, and 344 did not have a fracture.

The internal validation data set consisted of 195 patients from whom 498 radiographs (PA, lateral, and oblique when present) were available. 5 patients were excluded due to poor image quality (as decided by the 3 (orthopedic) trauma surgeons), making the final set 190 patients. The internal validation contained 47 patients with a 2R3A fracture, 45 2R3B, 53 2R3C and 45 without a fracture.

The external validation consisted of 200 patients, of which 12 patients were excluded due to poor image quality, making the final set 188 patients. The external validation data set contained 48 patients with a 2R3A fracture, 27 2R3B, 59 2R3C and 54 without a fracture. It consisted of a total of 376 radiographs (PA and lateral). The overall number of images in the validation and external validation data sets are based on comparative studies on the matter [1, 29, 30].

### Gold standard: surgeon interobserver agreement

Three (orthopedic) trauma surgeons independently classified each fracture in the internal and external validation sets. Using the results from the external validation, an inter-observer agreement was calculated. The classifications of each surgeon before any consensus meeting were used, including the option ‘exclude’ in case of perceived bad image quality. The overall inter-observer agreement was 0.65 (95%CI 0.60–0.69), often referred to as substantial agreement [31]. See Table 4 for the inter-observer agreement of each individual classification.

**Table 4:** Inter-observer agreement on the external validation set

Category	*Kappa*		*95% Confidence interval*	*p-value*
Overall	0.65		0.60—0.69	< 0.000
A	0.66		0.47—0.63	< 0.000
B	0.67		0.53—0.70	< 0.000
C	0.73		0.54—0.70	< 0.000
No Fracture	0.92		0.80—0.97	< 0.000
Exclude	0.00	−0.11—0.053		0.490

### CNN performance: internal validation

The algorithm's accuracy in classifying DRFs on the internal validation was 62%. The AUC for type 2R3A was 0.84, type 2R3B 0.63, type 2R3C 0.75, and patients with no DRF 0.93. Table 5 demonstrates the sensitivity and specificity of the algorithm. Removing the patients without a DRF did not improve results and are further specified in Table 6.

**Table 5:** Performance of the algorithm on classifying distal radial fractures

	Internal validation				External validation			
	Accuracy: 62%				Accuracy: 61%			
	*2R3A*	*2R3B*	*2R3C*	*No DRF*	*2R3A*	*2R3B*	*2R3C*	*No DRF*
AUC	0,84	0,63	0,75	0,93	0,82	0,56	0,75	0,88
Sensitivity	81%	27%	47%	96%	83%	15%	39%	89%
Specificity	78%	92%	95%	85%	74%	98%	75%	81%

**Table 6:** Performance of the algorithm on classifying distal radial fractures, after excluding patients without a DRF

	Internal validation			External validation		
	Accuracy: 52%			Accuracy: 50%		
	*2R3A*	*2R3B*	*2R3C*	*2R3A*	*2R3B*	*2R3C*
AUC	0,79	0,60	0,73	0,76	0,54	0,73
Sensitivity	81%	27%	47%	83%	15%	39%
Specificity	69%	89%	92%	61%	97%	91%

### CNN performance: external validation

The algorithm's accuracy in classifying DRFs on the external validation was 61%. The AUC for type 2R3A was 0.82, type 2R3B 0.56, type 2R3C 0.75, and patients with no DRF 0.88. Table 5 shows the sensitivity and specificity of the algorithm. Removing the patients without a DRF did not improve results and are further specified in Table 6.

### Prediction matrix

Two prediction matrices have been provided to accurately portray where the algorithm made mistakes in the classification of DRFs. See Tables 7 and 8 for the internal and external validation prediction matrix, respectively. For internal and external validation, most mistakes are made in 2R3C fractures being classified as 2R3A fractures by the algorithm and 2R3B fractures being missed as the algorithm predicted no fracture.

**Table 7:** Prediction matrix internal validation per patient. Shows the correct and incorrect prediction of the algorithm per classification. Bold numbers show the number of correctly predicted classifications

		Prediction given by algorithm			
		2R3A	2R3B	2R3C	No Fracture
Cla ssification	2R3A	**38**	2	5	2
	2R3B	11	**12**	2	20
	2R3C	19	9	**25**	0
	No Fracture	2	0	0	**43**

**Table 8:** Prediction matrix external validation per patient. Shows the correct and incorrect prediction of the algorithm per classification. Bold numbers show the number of correctly predicted classifications

		Prediction given by algorithm			
		2R3A	2R3B	2R3C	No Fracture
Classification	2R3A	**40**	1	2	5
	2R3B	6	**4**	5	12
	2R3C	27	2	**23**	7
	No Fracture	4	0	2	**48**

## Discussion

**Fig. 2:**
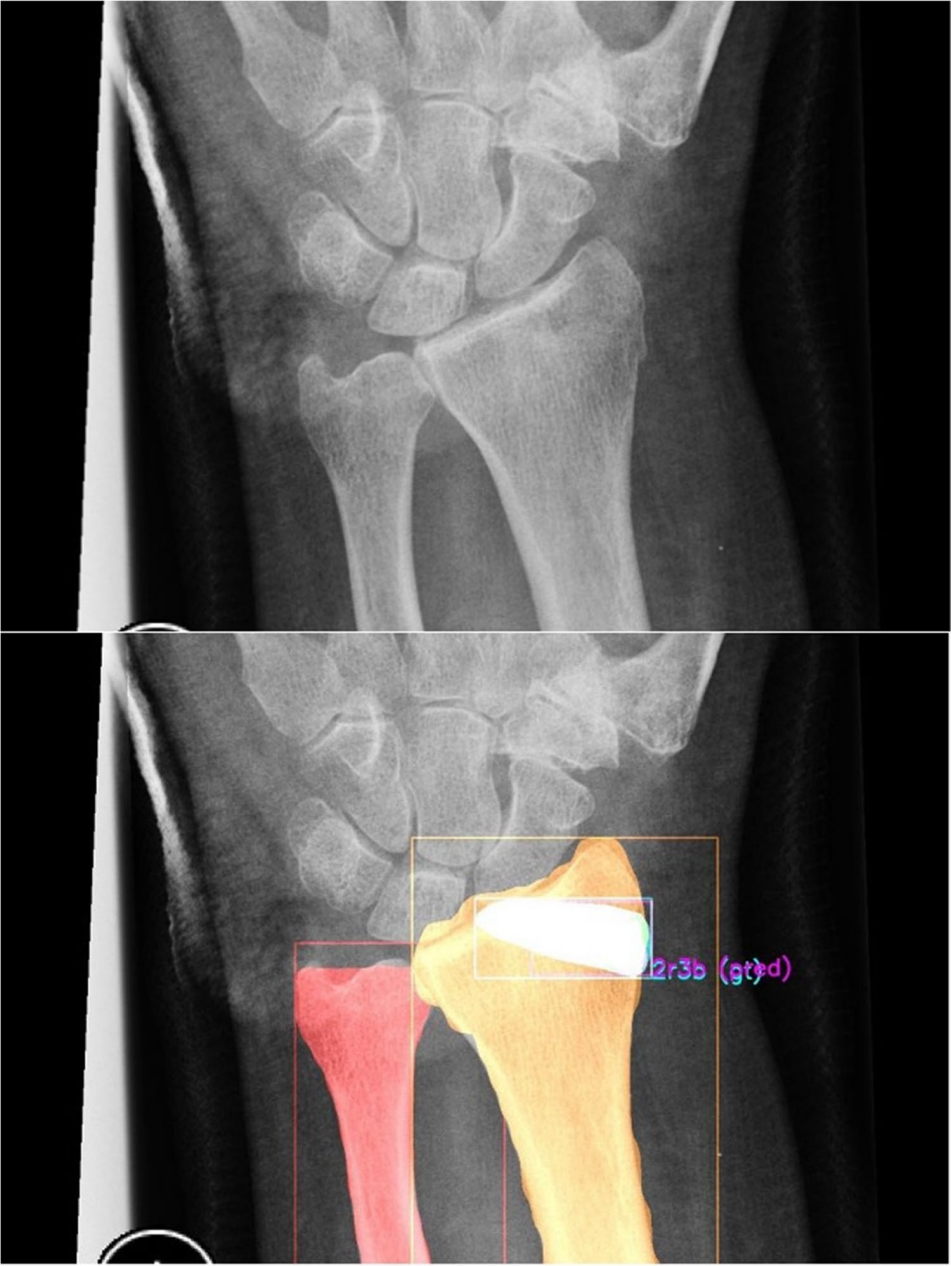
Above a distal radial fracture, underneath the output of the algorithm. The algorithm correctly classified a 2R3B fracture. The white outline (blue and purple overlap) shows that the prediction of the algorithm (purple) and the annotation by the researcher (blue) overlap. The algorithm also outlines the radius and ulna (orange and red respectively)

Classification of fractures should facilitate a practical discussion between healthcare professionals, not only in the treatment of patients but also in research. However, previous studies have shown poor inter- and intraobserver reliability for DRF classifications. The presented CNN algorithm has demonstrated excellent accuracy in classifying type 2R3A DRFs and excluding DRFs, and poor to moderate accuracy in classifying 2R3B and 2R3C DRFs according to the AO/OTA system, similar to surgeons (Fig. 2). Looking at the confusion matrix (Table 7 and 8), the algorithm classified 2R3B fractures as ‘no fracture’ and 2R3C mainly as 2R3A fractures. Identifying where this mix-up comes from is difficult through the ‘black box’ of the algorithm, but the algorithm clearly underestimates the fractures, rather than overestimating them in complexity.

Previously our research group has shown excellent results in detecting and localizing DRFs using a CNN algorithm with an AUC of 0.93, but similar results were not reproducible for classifying DRFs [4]. The poor reliability of classifying DRFs might be caused by the overlapping ulna and radius on lateral radiographs which can obstruct important features of the fracture, which is not the case in other fractures with good reliability such as hip fractures. The poor to moderate inter- and intra-observer reliability of both human and AI in classifying DRFs shows the inherent problem of the traditional classification systems, whereas looking at only extra- vs intra-articular fractures showed better reliability [9]. To overcome this struggle, we could try to find a new way of classifying which shows increased reliability.

There are several limitations to this study. First, the CNN algorithm was trained using a training set that excluded poor image quality, mainly when the distal radius was poorly visible. By excluding these lesser-than-ideal images, we created a selection bias. For the CNN algorithm to be more applicable in a real clinical setting, the algorithm should also be trained with these suboptimal radiographs. However, if the distal radius is obstructed in any way, it is difficult or impossible for either human or AI to diagnose and classify a fracture and would require new radiographs to be made in clinical practice. Second, the classification systems of the DRFs have poor reliability and reproducibility, as seen in the interobserver agreement between surgeons when classifying the validation sets [13–22]. Using CT scans to classify each fracture and then correlating the classification to each radiograph probably increases the quality of the validation sets and improves the golden standard. In addition, no further distinction was made between the subgroups within the AO/OTA classification. However, this could have worsened the interobserver agreement between the surgeons [20]. Third, the labeling and annotation process was performed by medical students. However, each radiograph was checked by a senior researcher under the supervision of an (orthopedic) trauma surgeon to prevent mistakes. Having experts label and annotate each radiograph might improve the algorithm, but this is very time intensive.

CNN algorithms in orthopedic trauma surgery has proven valuable in detecting and classifying fractures from plain radiography [32]. Several studies showed its CNN algorithm to be at least as capable as clinicians in fracture detection, and classification other than DRFs [1–3, 5]. In addition, Lindsey et al. showed promising results of the clinical applicability of a DRF-detecting algorithm by significantly improving the diagnostic accuracy of the clinician while being aided by the algorithm [30]. Two studies have assessed a CNN algorithm’s performance in classifying DRFs, although not using traditional classification systems and with mixed results [9, 10]. Min et al., who looked at extra- vs intra-articular fractures showed an AUC of 0.82, similar to our algorithm in classifying 2R3A DRFs and excluding fractures. Toblet et al., looked more in detail at fragment displacement, joint involvement and multiple fragments. Their AUC ranged from 0.59–0.92, more similar to our results. Interestingly, Min et al. reached an accuracy of 81% on detecting joint surface involvement, whereas Tobler et al. reached 63.7% accuracy. Our algorithm classified extra- vs intra-articular fractures (2R3A vs 2R3B and 2R3C) in 70% and 67% accuracy, on the internal and external set respectively, but results might have been better if trained specifically for that purpose. Unfortunately excluding patients without DRFs did not help to improve the accuracy of the algorithm. In future research, we could train a different algorithm using just radiographs with a DRF to improve accuracy. Allowing the algorithm to focus purely on classifying fractures, without the added difficulty of determining whether there is a fracture or not, might improve accuracy. However, the current algorithm is clinically more applicable by allowing radiographs of all painful wrists after trauma with a suspected DRF.

Our algorithm was very pragmatic with chances of high clinical performance, as it showed only slightly decreased performance on patients on the other side of the world. The created algorithm is made freely available to the public, allowing other researchers to further improve and test the algorithm. This provides insights into both the algorithm's practical applications and the impact of scaling patient numbers from different hospitals on its accuracy. Data has yet to be available on this. The algorithm can also show where it believes the fracture is, and outline the radius and ulna. This will make verifying the algorithm easy. If the algorithms accuracy increases, further research can be done to add AO/OTA classification subtypes, making the algorithm applicable in areas where more detail is wanted.

In conclusion, the algorithm has demonstrated moderate accuracy in classifying DRFs according to the AO/OTA system, similar to surgeons. These results show that despite previous excellence in fracture detection, CNN-algorithms struggle with classifying DRFs; potentially showing the inherent problems with these classification systems. Other centers are able to use this algorithm by training it or performing an external validation themselves.

## Data Availability

The code has been made publicly available for further training or external validation on GitHub (https://github.com/AIML-MED/DRF_Classification_Public).
